# Role of ultrasonography in diagnosing early rheumatoid arthritis and remission of rheumatoid arthritis - a systematic review of the literature

**DOI:** 10.1186/ar4132

**Published:** 2013-01-08

**Authors:** David F Ten Cate, Jolanda J Luime, Nanno Swen, Andreas H Gerards, Mike H De Jager, Natalja M Basoski, Johanna MW Hazes, Cees J Haagsma, Johannes WG Jacobs

**Affiliations:** 1 Rheumatology, Erasmus Medical Center Rotterdam, Dr. Molewaterplein 50-60, Rotterdam, 3015 GE, The Netherlands; 2 Rheumatology, Medical Center Alkmaar, Wilhelminalaan 12, Alkmaar, 1815 JD, The Netherlands; 3 Rheumatology, Vlietland Hospital Schiedam, Vlietlandplein 2, Schiedam, 3118 JH, The Netherlands; 4 Rheumatology, Albert Schweitzer Hospital, Albert Schweitzerplaats 25, Dordrecht, 3318 AT, The Netherlands; 5 Rheumatology, Maasstad Hospital, Maasstadweg 21, Rotterdam, 3079 DZ, The Netherlands; 6 Rheumatology, Hospital Group Twente, Zilvermeeuw 1, Almelo, 7609 PP, The Netherlands; 7 Rheumatology & Clinical Immunology, University Medical Center Utrecht, Heidelberglaan 100, Utrecht, 3584 CX, The Netherlands

## Abstract

**Introduction:**

Ultrasonography (US) might have an added value to clinical examination in diagnosing early rheumatoid arthritis (RA) and assessing remission of RA. We aimed to clarify the added value of US in RA in these situations performing a systematic review.

**Methods:**

A systematic literature search was performed for RA, US, diagnosis and remission. Methodological quality was assessed; the wide variability in the design of studies prohibited pooling of results.

**Results:**

Six papers on the added value of US diagnosing early RA were found, in which at least bilateral metacarpophalangeal (MCP), wrists and metatarsophalangeal (MTP) joints were scanned. Compared to clinical examination, US was superior with regard to detecting synovitis and predicting progression to persistent arthritis or RA. Eleven papers on assessing remission were identified, in which at least the wrist and the MCP joints of the dominant hand were scanned. Often US detected inflammation in patients clinically in remission, irrespective of the remission criteria used. Power Doppler signs of synovitis predicted X-ray progression and future flare in patients clinically in remission.

**Conclusions:**

US appears to have added value to clinical examination for diagnosing of RA when scanning at least MCP, wrist and MTP joints, and, when evaluating remission of RA, scanning at least wrist and MCP joints of the dominant hand. For both purposes primarily power Doppler US might be used since its results are less equivocal than those of greyscale US.

## Introduction

The treatment of rheumatoid arthritis (RA) has improved dramatically over past decades with the early and intensive use of conventional disease modifying antirheumatic drug (DMARD) strategies [1] and the introduction of biological agents [2]. Treatment strategies with dose and medication adjustments tailored to the individual patient (tight control) to achieve a predefined level of low disease activity, or preferentially, remission within a certain limited period of time (treat to target) [3] are nowadays used for early RA [4]. It is widely accepted that early after the onset of RA, there is a period of time (window of opportunity) during which effective treatment can beneficially alter the outcome of the disease in the long term [5,6]. This requires prompt referral and recognition of RA. Recently, new classification criteria [7] and new remission criteria [8] have been published. In the new classification criteria, it is suggested that imaging techniques such as ultrasonography (US) may be used for additional information in joints clinically suspected of arthritis [7]. Regarding remission criteria, a considerable number of patients in clinical remission according to several clinical criteria shows signs of inflammation on US [9-11]. These findings imply that US may have added value to clinical examination when diagnosing RA, or evaluating remission in RA. For this purpose we would have to make a selection of joints to evaluate by US, because assessment of all joints would be very time consuming.

The aim of this systematic review is to clarify if US, when used for diagnosing RA and for evaluating remission in RA, would give additional information to clinical examination, to elucidate which minimal set of joints should be assessed by US for these two purposes, and by which modality, that is, power Doppler US (PDUS) and/or greyscale US (GSUS).

## Material and methods

A systematic literature search was performed in PubMed, Embase and the Cochrane library for articles published up to October 4, 2011. A list of relevant keywords and synonyms for disease (RA, arthritis) and imaging (ultrasonography) was compiled. Keywords, including words of the title and abstract, and medical subject headings (Mesh) were combined using Boolean operators (AND, OR) (see Additional file 1). Included studies were those on adult humans, published in the English or Dutch language, either on diagnosing RA or evaluating US signs of synovitis in RA patients who were clinically in remission. We limited our study to the signs of US inflammation and did not assess structural joint damage. In the domain of diagnosis, assessing structural change probably would not increase the additive value of US very much, given the already very sensitive current 2010 American College of Rheumatology (ACR) classification criteria. Second, there would have been the problem of how to apply the US finding of structural damage to the 2010 ACR classification criteria. In the domain of remission, structural changes are a sign of past inflammation only. Disagreements about study inclusion were resolved by discussion; results are based on full consensus. Excluded were reviews, editorials, case reports and letters to the editor. One reviewer (DTC) screened titles and abstracts. Relevant articles were obtained and their reference lists were screened to find additional studies. Data were extracted by one reviewer (DTC) on year of publication, study population, study design and duration, treatment, possible follow up, number and type of joints under investigation, statistical methods and US parameters.

We used an adaptation of the phases (levels) in diagnostic studies proposed by Sackett and Haynes to reflect the clinical relevance of research data (Additional file 2, box 1) [12]. All results were summarized descriptively. Heterogeneity in study design and methods precluded pooling the results. Methodological quality of studies on diagnosing RA was assessed by the instrument Quality Assessment of Diagnostic Accuracy Studies-2 (QUADAS-2) [13], with an extra question on sample size. For the studies evaluating remission a quality assessment tool was not available; we created a quality items list (Additional file 3). The quality assessments were performed to check for possible flaws in study design and analyses.

## Results

### Diagnosing RA

In our systematic search we found six papers on the added value of US joint core sets in diagnosing RA [14-19]. All studies can be considered phase three, according to Sackett and Haynes. Arthritis was evaluated using both GSUS and PDUS. For definitions of US signs of inflammation see Table 1. In four of these studies wrists and metacarpophalangeal (MCP) joints were assessed as the minimum [14-17]; study-one also evaluated tendons [14], study-two also evaluated proximal interphalangeal (PIP) joints [15], and the third study extended the core set to metatarsophalangeal (MTP) joints and larger joints, such as the shoulders, knees and ankle [16]. The fourth study also included the distal interphalangeal (DIP) and elbow joints [17]. In the fifth study [18], painful joints and the adjacent joints of the same joint region (if applicable) and their contralateral joints were assessed, whereas the sixth study started scanning painful joints only; during this study the protocol was changed to US of MCP joints, MTP joints and knees bilaterally [19].

**Table 1:** Value of ultrasonography in diagnosing early (rheumatoid) arthritis

Author (year) [ref] Phase*	Study population	Number; type of joints or tendons	US modality, definition of synovitis (A-F)	Outcome, Risk estimates (95% CI)	Authors' conclusions	Limitations^#^
Filer (2011) [16] 3	58 IA 22 RA	38; MCP, PIP, MTP, wrists, elbows, shoulders, knees, ankles	GSUS & PDUS A	US reclassifies 3 of 29 IA patients to RA (ACR 2010)	US of MCP, wrists and MTP improves optimally clinical models to predict RA	Q4E
Freeston (2010) [14] 3	30 patients with inflammatory symptoms and negative RF and aCCP tests	12; MCP, wrists and flexor tendons of fingers	GSUS & PDUS B	US increases pretest probability for arthritis at one year from 6 to 94%	Inflammation at US predicts development of arthritis in patients with inflammatory symptoms and negative RF and aCCP tests	Q1D, Q2C
Ozgul (2009) [17] 3	51 IA	50; shoulders, elbows wrists, MCP, PIP, DIP, knees, ankles, MTP	GSUS C	Kappa = 0.61 for US diagnosis of RA versus clinical diagnosis	Average agreement between US and clinical examination when diagnosing RA	Q1A, Q1E, Q4E
Salaffi (2010) [15] 3	149 UA	18; wrists, MCP2-5, PIP2-5	GSUS & PDUS D	Sensitivity 0.35 (0.24, 0.48) and specificity 0.78 (0.67, 0.86) for US as baseline test, predicting RA at 1 year	Presence of PDUS signs predicts progression from UA to RA	Q1A, Q1C, Q1E, Q3B, Q4E
Stadt (2010) [18] 3	192 RF and/or aCCP-positive arthralgia patients, clinically without arthritis	Painful joint + adjacent and contralateral joints, mean 8 joints per patient	GSUS & PDUS E	JE: OR = 3.07 (1.05, 8.9); SH: OR = 5.5 (2.3, 12) PD: OR = 5.5 (2.6, 12), all for future arthritis	Inflammation at US predicts future arthritis in aCCP-positive patients clinically without arthritis	Q4E
Wakefield (2004) [19] 3	80, Oligoarthritis	First 40 patients: only symptomatic joints Last 40 patients: 22; MTP, MCP, Knees	GSUS F	1/3 of cases of oligoarthritis reclassified to polyarticular (>= 6 joints) arthritis	GSUS detects inflammation in more joints than clinical exam, but 8% of joints with clinical synovitis are normal at US	Q2C

A, according to Szkudlarek 2003 [30], 2006 [31] and Wakefield 2005 [32]. B, semiquantitative definitions based on binary definitions by Brown 2006 [11], Wakefield 2004 [19], Karim 2004 [33] and Newman 1996 [34]. C, (a) joint capsule elevated beyond normal range, or (b) synovial hypertrophy or effusion around the joints, or (c) erosions of the joint margins, or (d) change in the diameter of the tendon with or without peritendinous hypo-echogeneity supporting effusion. D, according to Wakefield 2005 [32] and Szkudlarek 2003 [30]. E, according to Szkudlarek 2003 [30]. F, abnormally hypo-echoic joint space, distinct from the intra-articular fat pad and non-compressible with the transducer. Ref, reference; US, ultrasonography; GSUS, greyscale ultrasonography; PDUS, power Doppler ultrasonography; aCCP, anti-cyclic citrullinated peptide; JE, joint effusion; IA, inflammatory arthritis; RA, rheumatoid arthritis; UA, undifferentiated arthritis; RF, rheumatoid factor; SH, synovial hypertrophy; OR, odds ratio. *Adaptation of phases according to Sackett and Haynes (Additional file 2, box 1): Phase 1. Do US results in patients with the condition differ from those without the condition? Phase 2. Are patients with certain US results more likely to have the condition? Phase 3. Do US results distinguish patients with and without the condition among those in whom it is clinically sensible to suspect the condition? Phase 4. Do patients undergoing US fare better in their ultimate health outcome than similar untested patients?. ^#^See Additional file 3.

At the joint level, among anti-cyclic citrullinated peptide (aCCP)-positive arthralgia patients (that is, those having no clinically swollen joints) US predicted progression to clinically detectable joint inflammation of the subset of joints showing a positive PDUS signal, after a median follow up of 26 months, with an odds ratio (OR) of 5.50 (95% CI 2.57, 11.9) [18]. Another study showed that adding US parameters at baseline to clinical parameters increased the pretest probability of 6% to 94% post test for the progression to inflammatory arthritis at 12 month follow up at patient level. This was evaluated among 30 rheumatoid factor (RF) and/or aCCP-negative individuals with inflammatory hand symptoms with or without clinical synovitis [14]. Among individuals with possible RA (*n *= 58), 10% (three out of twenty-nine patients) were rightly classified as RA patients by US at baseline, using the clinical diagnosis of RA at 1.5 years as the reference standard [16]. In 80 patients with early oligo-arthritis(< 12 months), about 1/3 of patients could be reclassified as having > 5 inflamed joints when US was added to the clinical examination, but 15 of all 185 joints (8%) with clinical synovitis were normal on US examination [19]. This is why the study extended its scan protocol halfway through the study, from scanning only painful joints to scanning MCP and MTP joints and knees bilaterally. In one study in which the shoulders, elbows, wrists, MCP joints, PIP joints, DIP joints, knees, ankles and MTP joints were scanned, among 51 inflammatory arthritis patients, the subgroup with US symmetric polyarthritis was compared with the subgroup, who two years thereafter, met the ACR 1987 criteria for RA, yielding a kappa statistic of 0.61, which denotes a reasonable level of agreement [17].

In another study US at baseline reclassified 15% (*n *= 22) of the patients with undifferentiated arthritis (*n *= 149) to having RA, using the clinical diagnosis of RA (*n *= 62) with a follow up of 12 months as reference, while US in 11% (*n *= 17) was false positive at baseline. For this study, as the cut-off for US inflammation, a PDUS signal in more than three joints was used [15]. This suggest a US sensitivity of 0.35 (95% CI 0.24, 0.48) and a specificity of 0.78 (95% CI 0.67, 0.86) for diagnosing RA at baseline.

#### Quality assessment of studies reviewed for diagnosing RA

Details of quality assessment of studies are shown in Table 1 and Additional file 3. Assessment of the methodological quality using the QUADAS-2 [13], extended with a question on sample size, showed relevant patients in all six studies, who were followed over time in five studies [15,16,18]. Both the index test (US) and reference (diagnosis of RA) were clearly described and applied to all included patients, although semiquantitative definitions of US inflammation used in one paper eventually seem to be based on a paper using a binary score and a paper describing synovitis of the knee [14]. Drop out of patients was mentioned in one of five longitudinal studies [14].

Furthermore, in our interpretation small sample sizes and heterogeneity of studies diminished the strength of evidence of the value of US to improve early diagnosis of RA [14-17].

### Evaluation of remission of RA

Our systematic search yielded 11 papers on the added value of US in the evaluation of remission in RA [9-11,20-27], using sets of joints ranging from six [10] to forty-four joints [26]. One study scanned forty-four joints [26], two studies scanned forty-two [20,27], and the other studies assessed between six and sixteen joints [9-11,21-25]. Within the 11 studies, the wrist and MCP joints of the dominant hand were always scanned. Arthritis was evaluated by GSUS and PDUS. The definition of remission varied and included physician-determined remission [11,20,21], a disease activity score (DAS) <1.6 [23,26], and complete absence of clinical and laboratory symptoms [9], while the time since remission varied from 2 months [9,20,22] to 3 months [26], or to more than 6 months [10,11,21,23-25]. One study evaluated the time to remission in a treatment setting [27]. Study characteristics, definitions of US signs of inflammation and outcomes are presented in Table 2.

**Table 2:** Value of ultrasonography in remission of rheumatoid arthritis

Author (year)[ref] Phase*	Study population, duration of remission or LDA (definition)	Number; type of joints	US modality, definition of synovitis (A-F)	Outcome^¶ ^Risk estimates (95% CI)	Authors' conclusions	Limitations^#^
Balsa (2010) [20] 2	97 RA in remission >2 months (physician's judgment); 88 controls	42; PIP, MCP, wrists, elbows, shoulders, knees, ankles, MT, MTP	GSUS & PDUS A	Of RA in remission, 92% GSUS-positive and 42% PDUS-positive; 88% of non-arthritic controls GSUS-positive	Of sets ARA 1981, DAS28 and SDAI, the SDAI is closest to the absence of inflammation on US	j,o

Brown (2006) [11] 2	107 RA in remission > 6 months (physician's judgment)	8; MCP2-5, wrist (RC, IC, DRU, UC) of dominant hand	GSUS & PDUS B	73% GSUS-positive 43% PDUS-positive	In many patients in remission, inflammation is found on US	o,x

Brown (2008) [21] 2	102 RA in remission > 6 months (physician's judgment)	8; MCP2-5, wrist (RC, IC, DRU, UC) of dominant hand	GSUS & PDUS B	PDUS predicts radiographic progression at one year: OR = 12 (3.3, 44)	Presence of PDUS predicts radiographic progression in joints of patients in remission	o,v,x

Foltz (2011) [22] 2	85 RA in remission or with LDA > 2 months (DAS < 2.4)	14; wrists, MCP2,3,5, MTP2,3,5	GSUS & PDUS A	PDUS predicts flare: OR = 6.3 (2.0, 20); PDUS predicts radiographic progression: OR = 1.4 (1.1, 1.9)	Inflammation on PDUS predicts radiographic progression and flare of patients in remission or with LDA	o,q,v

Ozgocmen (2008) [9] 1	52 RA of whom a subgroup in remission > 2 months (Preliminary ARA 1981 + DAS < 3.2)	14; MCP1-5, wrists (USTL, RSTL)	GSUS & PDUS C	62% of 32 patients (in ARA 1981 remission) PDUS-positive 58% of 31 patients (in DAS28 remission) PDUS-positive	PDUS inflammation is found for 2 different remission criteria sets, in a similar number of patients	i,j,k,l,o,r,t,

Peluso (2011) [23] 2	48 early (7 months) RA in remission > 6 months (DAS < 1.6) 46 LSRA (118 mo.) in remission > 6 months (DAS < 1.6)	12; MCP2-3, PIP2-3, wrists (RC, UC)	GSUS & PDUS Not given	56% early RA GSUS-positive; 83% LSRA GSUS-positive; PDUS predicts flare: OR = 3.6 (1.4, 9.0)	PDUS shows predictive value for flare In many patients in remission, inflammation is found on US	g,i,l,n,o,p,r,t,u,v

Saleem (2009) [24] 2	100 RA in remission > 6 months (DAS < 2.6)	16; wrists (RC, UC,IC), MCP1-5	GSUS & PDUS D	88% GSUS-positive PDUS-positive 58%	In > 55% of patients in remission inflammation is found on PDUS	i,o,r,x

Saleem (2010) [25] 2	47 RA in remission > 6 months (DAS28 < 2.6) of whom a subgroup of 20 patients is assessed also by US	18; wrists, PIP, MCP	GSUS & PDUS F	In group that flared after 2 years, at baseline 88% GSUS-positive and 44% PDUS-positive. In group that did not flare after 2 years, at baseline 82% GSUS-positive and 45% PDUS- positive	In many patients in remission inflammation is found on PDUS, but does not predict flare	o,r,u,v,w,x

Saleem (2011) [10] 2	128 RA in remission > 6 months (ACR 2011)	6; wrist, MCP2-5 of the dominant hand	GSUS & PDUS D	Around 50% PDUS-positive when using ACR2011, DAS28, SDAI remission sets	For 3 different remission criteria sets a similar number of patients is PDUS-positive	c,h,i,j,k,l,o,p,r,t,u,v,x

Scire (2009) [26] 2	106 early RA (<12 months), of whom 43 in remission > 3 months (DAS < 1.6)	44; shoulders, elbows, wrists, MCP, PIP, knees, ankles, MTP	GSUS & PDUS E	95% GSUS-positive, 41% PDUS-positive PDUS positivity predicts flare: OR = 13 (1.6, 104)	In many patients in remission, inflammation found on PDUS, predictive of flare	g,i,j,k,l,m,p,r,t,u,v

Wakefield (2007) [27] 3	10 early (<12 months) RA in remission (DAS28 < 2.6)	42; shoulders, elbows, wrists, MCP, PIP, knees,tibiotalar, MT, MTP	GSUS & PDUS Not given	50% GSUS-positive and 15% PDUS-positive in clinically normal joints	In many RA-patients in remission, PDUS inflammation is found	i,n,t,x

A, according to Wakefield 2005 [32]. B, semi quantitative definitions based on binary definitions by Brown 2006 [11], Wakefield 2004 [19], Karim 2004 [33] and Newman1996 [34]. C, GSUS: Hypoechogeneity; PDUS: grade 0, no flow signal in the synovium; grade 1, separate dot signals or short linear signals; grade 2, clearly discernible vascularity with either many small vessels or several long vessels with or without visible branching, though involving less than half the area of the synovium; grade 3, vessels involving more than half the area of the synovium. D, semiquantitative definitions based on binary definitions by Brown 2006 [11], Wakefield 2004 [19], Karim 2004 [33]. No PDUS definitions. E, Wakefield 2005 [32] and Newman 1996 [34]. F, according to Outcome Measures in Rheumatology (OMERACT) definitions, but not clear from footnotes which definitions have been used. US, ultrasonography, GSUS, greyscale ultrasonography; PDUS, power Doppler ultrasonography; aCCP, anti-cyclic citrullinated peptide; JE, joint effusion; SH, synovial hypertrophy; IA, inflammatory arthritis; LDA, Low disease activity; LSRA, long standing RA; RA, rheumatoid arthritis; UA, undifferentiated arthritis; RF, rheumatoid factor; OR, odds ratio; DRU, distal radio-ulnar; IC, intercarpal; MT, midtarsal; RC, radiocarpal; RSTL, radial styloid; USTL, ulnar styloid; UC, ulnocarpal; SDAI, simplified disease activity index. *Adaptation of phases according to Sackett & Haynes (Additional file 2, box 1): Phase 1. Do US results in patients with the condition differ from those without the condition? Phase 2. Are patients with certain US results more likely to have the condition? Phase 3. Do US results distinguish patients with and without the condition among those in whom it is clinically sensible to suspect the condition? Phase 4. Do patients undergoing the US fare better in their ultimate health outcome than similar untested patients? # See Additional file 3. ¶GSUS pos. (%) and PDUS pos. (%) signify the percentages of individuals with at least one inflamed joint applying the respective US modality.

In all 11 papers, there was a discrepancy between the number of clinically swollen joints and the higher number of joints with US signs of arthritis, indicating that joints that were not clinically inflamed showed US signs of arthritis. In five of these eleven papers it was explicitly mentioned that US evidence of synovitis was also found in joints that were not clinically inflamed [10,11,23,24,26]. There seemed to be no clear association between the number of joints scanned per patient and the number of patients with at least one joint with US signs of synovitis. When looking at GSUS signs of synovitis, all 11 studies identified synovitis in 73 to 95% of patients in clinical remission; for PDUS signs of synovitis, the range was 8.7 to 62% [9-11,20-27].The predictive ability of US for clinical flares was evaluated in four studies [22,23,25,26]. Three of these identified predictive value, where one did not [25]. One study reported an OR of 3.6 (95% CI 1.4, 9.0) for the occurrence of flare in PDUS-positive patients when scanning the wrists, and the second and third MCP and PIP joints, all bilaterally [23]. In another study, PDUS signs of synovitis were associated with an OR of 6.3 (95%CI 2.0, 20) [22] for the occurrence of flare within one year among patients in clinical remission (DAS44 < 2.4) when scanning the wrists, and the second, third and fifth MCP and MTP joints, all bilaterally. In a study assessing 44 joints among patients in remission, PDUS signs of synovitis predicted flare with an OR of 13 (95%CI 1.6,104) [26]. The predictive value of GSUS was either not significant [22,25,26] or not presented [23].

Two of the eleven papers evaluated progression of radiological joint damage in patients in clinical remission. The presence of PDUS signs of inflammation increased the risk of joint damage with an OR of 1.4 (95%CI 1.1, 1.9) at the patient level in a study of nine patients with radiographic signs of progression [22]. At the joint level, presence of PDUS signs of inflammation predicted progression with an OR of 12 (95%CI 3.3, 44) in a study of 10 patients [21]. GSUS scores were significantly higher in the group that progressed vs. the group that did not progress (mean 4.8 ± SD 2.3 vs. 3.2 ± 2.6) [22], or they predicted radiographic progression with an OR of 1.92 (95% CI 0.49, 7.24) [21].

The impact of using different remission criteria was reported in five studies. Two of these studies presented discrepancies between the prevalence of inflammation detected by US if applying different remission criteria. The first of these two studies showed that among patients in remission according to the simplified disease activity index when using a cutoff point of less than 3.3 (SDAI score < 3.3), the number of joints with PDUS signs of synovitis was smaller, and the PDUS grade of synovitis was lower when compared to those of patients in remission when using a cutoff point of less than 5.5 on the SDAI (SDAI score < 5.5), the DAS28 (cutoff score < 2.4 or < 2.6) or slightly modified ACR 1981 remission criteria (that is, excluding the fatigue criterion). In this study 42 joints were scanned [20]. The second of these two studies showed that using the ACR 1981 remission criteria, the number of patients with US inflammation was smaller compared to applying the criterion of a DAS < 1.6 [23]. In this study 12 joints in the hands and wrists were scanned. The three other studies reporting on the impact of using different remission criteria showed that 60 to 80% of their patients had GSUS signs of inflammation independently of the specific criteria used [9-11]. Regarding PDUS signs of inflammation, two of these three studies showed that these signs were present in about 50% of the patients in clinical remission when scanning the MCP joints and the wrist of the dominant hand [10,11]. The third study, in which a greater number of joints was scanned, showed similar results: about 60% of the patients in clinical remission showed, irrespective of the clinical remission criteria used, PDUS signs of inflammation when scanning the wrists (ulnar and radial styloid regions) and the first to fifth MCP joints, all bilaterally [9].

The influence of disease duration was studied in one paper, assessing 12 joints in the hands and wrists. Among patients who were in clinical remission, 44% of those with early RA had no US signs of synovitis (defined as absence of GSUS and PDUS signs of inflammation) vs. 17% of those with longstanding RA. When defining synovitis as presence of GSUS synovitis and absence of PDUS synovitis, 15% of those with early RA had US signs of synovitis, as opposed to 52% of the patients with longstanding RA. When defining synovitis as presence of both GSUS and PDUS signs of inflammation, 42% of the patients with early RA showed signs of US inflammation, as opposed to 30% of those with longstanding RA [23].

#### Quality assessment of studies reviewed for evaluation of remission of RA

Details of quality assessment are shown in Table 2 and Additional file 3. Quality assessment was performed for all studies but longitudinal studies are the most relevant to evaluate the remission of RA. Two of these studies evaluated the added value of US for prediction of radiographic progression among patients in remission, and four studies evaluated the value for prediction of flare. We found wide CIs and point estimates differing from study to study, probably due to small sample sizes, slightly different definitions for remission and flare and analyses performed at joint level and at patient level. Definitions of US inflammation are not clearly described in three of the eleven papers [23,25,27]. In four other longitudinal studies the semiquantitative definitions of US inflammation used for inflammation in MCP joints and wrists seem to be based on a paper using a binary score and a paper describing synovitis of the knee [10,11,21,24].

## Discussion

The results of our systematic search indicate that when diagnosing RA a greater number of inflamed joints per patient was detected by US compared to clinical examination in populations ranging from aCCP/RF-positive patients with arthralgia, to patients with clinically observed arthritis. The presence of US signs of inflammation seems to increase the risk of progression to persistent arthritis or RA, implying clinical relevance. Regarding assessment of remission, our review shows that in many patients with low disease activity or in clinical remission, US signs of inflammation were detected, even in those who met stringent clinical remission criteria. These findings are relevant, because the results of these studies suggest that PDUS signs of synovitis predict progression of radiographic joint damage and flare. We limited our study to signs of US inflammation and did not assess structural joint damage. The reason for this is that in the publication of the new classification criteria, in which erosions are not included, it was suggested that US may be used to confirm clinical findings, i.e. swelling of the joint. Erosions typical of RA would imply the classification of RA in patients who met the new classification criteria in the past. However, the new classification criteria are very sensitive: the diagnosis of RA can be made on the basis of one swollen joint, so one could argue that for these new criteria, the finding of erosions would not add much to the sensitivity in early RA, in contrast to the situation with the 1987 criteria. Second, there would have been the problem of how to apply the finding of structural damage, assessed by US, to the 2010 ACR classification criteria, that is, what would be the contribution of structural damage assessed by US to the diagnosis, applying the 2010 criteria? In the domain of remission, structural damage reflects inflammation in the past, not the current inflammatory state.

An important question is, which joints should be scanned? Scanning only the joints that are painful or clinically show arthritis does not seem to be a valid strategy, and scanning all joints is not feasible in daily practice. Based on the spectrum of joints most frequently involved in early RA and the results of this review, a recommendation when scanning for diagnosis of early RA could be to scan at the minimum the wrists, MCP and MTP bilaterally using PDUS; PIP joints could be included based on the results of one study. Also in the domain of remission of RA it is important to identify which joints to scan. Although more signs of arthritis are found when scanning a larger number of joints, a clear relation between the number of joints scanned and the number of patients clinically in remission with US signs of synovitis seems lacking. Therefore, it might be sufficient to scan a limited set of joints for this purpose. In eleven studies the wrist and MCP joints of the dominant hand had been scanned as the minimum.

Based on the results of this review it seems that it is not necessary to scan large joints when diagnosing RA or evaluating the remission of RA. In general, the more joints that are scanned, the higher the chance of finding US signs of arthritis in a patient. An earlier diagnosis leads to earlier initiation of adequate therapy, more often within the window of opportunity. This not only improves the prognosis in the short term, for example, by inducing remission at an earlier stage and more frequently, but possibly it also favorably alters the long-term course of the disease.

Another important question is which modality to use, PDUS or GSUS? Our systematic search indicates that PDUS in particular may have an added value in the diagnosis of early RA and evaluation of the remission of RA: the predictive value of PDUS was higher than that of GSUS. This is in line with the findings that GSUS signs of inflammation also occur in non-arthritic individuals [20]. In a study in an osteoporosis outpatient clinic, GSUS signs of synovitis were detected in up to 88% of 16 individuals who were without clinical symptoms or signs of joint disease (controls), based on scanning 42 joints with a cutoff of at least one joint with a score of 1 according to the OMERACT criteria for synovial hypertrophy [20]. Of all 672 joints scanned, 76 joints showed GSUS signs of synovitis, 64 of them with grade 1, 12 with grade 2, and none with grade 3 signs. In another study, in which a total of 84 joints was scanned among nine healthy individuals, 23 joints showed GSUS grade 1 signs of inflammation and only one joint was scored grade 2; no joints had a grade 3 score [18]. It seems that for the purpose of discriminating arthritis patients from non-arthritic patients, the use of the GSUS grade 1 score is debatable. Also in RA patients it is not clear what the significance of GSUS grade 1 is. One study states that in longstanding RA, GSUS might depict chronically thickened tissue without inflammation [23]. At the patient level, a cumulative GSUS score for discriminating arthritis patients from non-arthritic patients has yet to be determined. A cutoff of 8 when scanning 22 joints has been proposed [28].

Although the predictive value of PDUS is higher than that of GSUS to predict early RA, flare of RA and radiographic progression, PDUS has limitations as well. It is a technique that is particularly operator-, machine- and setting-dependent [29]. It is important to avoid pressure on the transducer, and motion artifacts, and to use the correct US settings, for example, wall filter and pulse repetition frequency should be low when assessing joints.

Although the study results in our systematic review generally were not conflicting for either diagnosis or remission, some considerations need to be made. For instance, the number of diagnostic studies is currently limited, and only one study has focused on the ACR/European League Against Rheumatism (EULAR) 2010 criteria. Furthermore, regarding the quality of studies, the longitudinal studies looking at events (flares or radiographic progression) are small, causing a wide variation in the US risk estimates. Also, the variables that have been shown to be predictors of the diagnosis of RA or of remission, such as radiographic joint data and aCCP test results, have not all been taken into account. This might have inflated the added value of US. In addition, clear definitions for US signs of inflammation were not always given.

Something else to consider is that some of the papers reviewed are from the same group [10,11,19,21,24,25,27]. Data presented in these papers might not be independent of each other, with correlated results being biased in one or the other direction. However, the results from the studies in our review are based on different patient populations. Also, we did not find signs that this group may be evidently pro or contra US, such that it would affect their scientific integrity, especially since one of the papers from this group shows a lower predictive value of US compared to those in other reviewed papers. Large prospective longitudinal studies are necessary to evaluate the additional value of US in diagnosing RA, scanning joints and evaluating the predictive validity of other signs such as US-detected tenosynovitis.

## Conclusions

In conclusion, although further research is needed, PDUS has additional value to clinical examination both in improving early diagnosis of RA and establishing true RA remission. GSUS seems less specific. In the diagnostic process, studies suggest that as a minimum the wrist, MCP and MTP joints should be scanned bilaterally, while for remission, studies suggest that as a minimum the wrist and MCP joints of the dominant hand should be scanned.

## Abbreviations

aCCP: anti-cyclic citrullinated peptide; ACR: American College of Rheumatology; DAS: disease activity score; DIP: distal interphalangeal; DMARD: disease modifying antirheumatic drug; DRU: distal radio-ulnar; EULAR: European League Against Rheumatism; GS: greyscale; GSUS: greyscale ultrasonography; IA: inflammatory arthritis; IC: intercarpal; JE: joint effusion; LSRA: long standing RA; MCP: metacarpophalangeal; MT: midtarsal; MTP: metatarsophalangeal; OR: odds ratio; PD: power Doppler; PDUS: power Doppler ultrasonography; PIP: proximal interphalangeal; RA: rheumatoid arthritis; RC: radiocarpal; RF: rheumatoid factor; RSTL: radial styloid; SDAI: simplified disease activity index; SH: synovial hypertrophy; UA: undifferentiated arthritis; UC: ulnocarpal; US: ultrasonography; USTL: ulnar styloid.

## Competing interests

The authors declare that they have no competing interests.

## Authors' contributions

All authors participated in the conception and design of this study and interpretation of the data. DTC, JJL and JWGJ performed the data extraction and the quality assessment. All authors were involved in drafting the article or revising it critically for important intellectual content. All authors read and approved the final manuscript.

## Supplementary Material

Additional file 1 **Search strategies**. Both for the domain of early rheumatoid arthritis (RA) and remission a separate search strategy for PubMed and Embase is presented, as well as a flow chart. Click here for file

Additional file 2 **Adaptation of the diagnostic phases (levels) of Sackett and Haynes**. Adaptation of Sackett and Haynes phases (levels) of diagnostic questions in diagnostic studies to reflect the clinical relevance of the reviewed studies. Click here for file

Additional file 3 **Quality assessment lists**. For the domain of early rheumatoid arthritis (RA) we used the Quadas-2, with an extra question on sample size. For the studies evaluating remission, no quality assessment tool was available, therefore we created a quality items list to evaluate these studies. Click here for file
