# Supplementary material for: Role of ultrasonography in diagnosing early rheumatoid arthritis and remission of rheumatoid arthritis - a systematic review of the literature
Source: Arthritis Res Ther. 2013 Jan 8;15(1):R4. doi: 10.1186/ar4132 (PMC3672772; doi:10.1186/ar4132)
Supplement: Additional file 1 — Search strategies. Both for the domain of early rheumatoid arthritis (RA) and remission a separate search strategy for PubMed and Embase is presented, as well as a flow chart. [file ar4132-S1.DOC]

**Search strategies**

Early RA

Pubmed: (ultrasonography[mesh] OR ultrasonography[sh] OR ultrasono*[tw] OR echogr*[tw]) AND (synovi*[tw] OR arthrit*[tw] OR inflammatory joint condition*[tw] OR joint inflammation*[ti] OR joint inflammation*[tw] OR oligoarthrit*[tw] OR polyarthrit*[tw]) AND ((index*[tw]) OR (score*[tw]) OR (scori*[tw]) OR (count[tw]))

Embase: (inflammat* NEAR/3 joint*):de,ab,ti OR oligoarthrit*:de,ab,ti OR polyarthrit*:de,ab,ti OR 'arthritis'/exp OR arthritis OR synovi* OR arthrit*:de,ab,ti AND (ultrasono* OR echogr*) AND (index*:de,ab,ti OR score*:de,ab,ti OR scori*:de,ab,ti OR count*:de,ab,ti)

Number of articles in search

1700

233

41

5

Exclusion on title Exclusion on abstract Exclusion on full text

Remission

Pubmed: (ultrasonography[mesh] OR ultrasonography[sh] OR ultrasono*[tw] OR echogr*[tw]) AND (arthrit*[tw] OR inflammatory joint condition*[tw] OR joint inflammation*[ti] OR joint inflammation*[tw] OR oligoarthrit*[tw] OR polyarthrit*[tw] OR disease activity[tw] OR DAS) AND remission

Embase: (arthrit*:ti,ab,de OR (inflammat* NEAR/3 joint*):ti,ab,de OR oligoarthrit*:ti,ab,de OR polyarthrit*:ti,ab,de) OR (‘disease activity’:ti,ab,de) OR (DAS) OR (arthritis/exp OR arthrit*:ti,ab,de) AND (ultrasono* OR echogr*) AND remission

Number of articles in search

387

23

16

11

Exclusion on title Exclusion on abstract Exclusion on full text
