# Supplementary material for: Role of ultrasonography in diagnosing early rheumatoid arthritis and remission of rheumatoid arthritis - a systematic review of the literature
Source: Arthritis Res Ther. 2013 Jan 8;15(1):R4. doi: 10.1186/ar4132 (PMC3672772; doi:10.1186/ar4132)
Supplement: Additional file 2 — Adaptation of the diagnostic phases (levels) of Sackett and Haynes. Adaptation of Sackett and Haynes phases (levels) of diagnostic questions in diagnostic studies to reflect the clinical relevance of the reviewed studies. [file ar4132-S2.DOC]

For the evaluation of studies on diagnosing RA, the condition of level 1, 2 and 3 is the diagnosis RA. For studies evaluating remission, this condition is the presence of clinical remission, according to several definitions.

| Box 1: Adaptation of the phases (levels) of Sackett & Haynes of diagnostic questions in diagnostic studies  • Phase 1. Do signs of US inflammation in patients with the condition differ from those without the condition?  • Phase 2. Are patients with certain signs of US inflammation more likely to have the condition?  • Phase 3. Do signs of US inflammation distinguish patients with and without the condition among those in whom it is clinically sensible to suspect the condition?  • Phase 4. Do patients undergoing the US fare better in their ultimate health outcome than similar untested patients? |
| --- |
