# Supplementary material for: Role of ultrasonography in diagnosing early rheumatoid arthritis and remission of rheumatoid arthritis - a systematic review of the literature
Source: Arthritis Res Ther. 2013 Jan 8;15(1):R4. doi: 10.1186/ar4132 (PMC3672772; doi:10.1186/ar4132)
Supplement: Additional file 3 — Quality assessment lists. For the domain of early rheumatoid arthritis (RA) we used the Quadas-2, with an extra question on sample size. For the studies evaluating remission, no quality assessment tool was available, therefore we created a quality items list to evaluate these studies. [file ar4132-S3.DOC]

**Quality assessment lists**

Quadas-2, with extra question on sample size.

**DOMAIN 1: PATIENT SELECTION**

| **A. Risk of Bias** | |
| --- | --- |
| Describe methods of patient selection: | |
| **Q1A** Was a consecutive or random sample of patients enrolled? | Yes/No/Unclear |
| **Q1B** Was a case-control design avoided? | Yes/No/Unclear |
| **Q1C** Did the study avoid inappropriate exclusions? | Yes/No/Unclear |
| **Q1D** Was the sample size appropriate? | Yes/No/Unclear |
| **Q1E Could the selection of patients have introduced bias?** | **RISK: LOW/HIGH/UNCLEAR** |
| **B. Concerns regarding applicability** | |
| Describe included patients (prior testing, presentation, intended use of index test and setting)**:** | |
| **Q1F Is there concern that the included patients do not match the review question?** | **CONCERN: LOW/HIGH/UNCLEAR** |

**DOMAIN 2: INDEX TEST(S)**

**If more than one index test was used, please complete for each test.**

| **A. Risk of Bias** | |
| --- | --- |
| Describe the index test and how it was conducted and interpreted: | |
| **Q2A**Were the index test results interpreted without knowledge of the results of the reference standard? | Yes/No/Unclear |
| **Q2B** If a threshold was used, was it pre-specified? | Yes/No/Unclear |
| **Q2C Could the conduct or interpretation of the index test have introduced bias?** | **RISK: LOW /HIGH/UNCLEAR** |
| **B. Concerns regarding applicability** | |
| **Q2D Is there concern that the index test, its conduct, or interpretation differ from the review question?** | **CONCERN: LOW /HIGH/UNCLEAR** |

**DOMAIN 3: REFERENCE STANDARD**

| **A. Risk of Bias** | |
| --- | --- |
| Describe the reference standard and how it was conducted and interpreted: | |
| **Q3A** Is the reference standard likely to correctly classify the target condition? | Yes/No/Unclear |
| **Q3B** Were the reference standard results interpreted without knowledge of the results of the index test? | Yes/No/Unclear |
| **Q3C Could the reference standard, its conduct, or its interpretation have introduced bias?** | **RISK: LOW /HIGH/UNCLEAR** |
| **B. Concerns regarding applicability** | |
| **Q3D Is there concern that the target condition as defined by the reference standard does not match the review question?** | **CONCERN: LOW /HIGH/UNCLEAR** |

**DOMAIN 4: FLOW AND TIMING**

| **A. Risk of Bias** | |
| --- | --- |
| Describe any patients who did not receive the index test(s) and/or reference standard or who were excluded from the 2x2 table (refer to flow diagram):  Describe the time interval and any interventions between index test(s) and reference standard: | |
| **Q4A**  Was there an appropriate interval between index test(s) and reference standard? | Yes/No/Unclear |
| **Q4B**  Did all patients receive a reference standard? | Yes/No/Unclear |
| **Q4C**  Did patients receive the same reference standard? | Yes/No/Unclear |
| **Q4D**  Were all patients included in the analysis? | Yes/No/Unclear |
| **Q4E**  **Could the patient flow have introduced bias?** | **RISK: LOW /HIGH/UNCLEAR** |

**Quality assessment remission:**

| **Quality assessment*** | |
| --- | --- |
| **Population** | |
|  | *Item* |
| **a** | Number of patients |
| **b** | Age, (mean/median +- SD/range) |
| **c** | Sex; (mean/median +- SD/range) |
| **d** | time since diagnosis; (mean/median +- SD/range) |
| **e** | time since remission; (mean/median +- SD/range) |
| **f** | therapy used at moment of remission |
| **g** | medication changes during period of remission |
| **h** | DAS(28) (mean/median +- SD/range) |
| **i** | Baseline erosions |
| **j** | HAQ(mean/median +- SD/range) |
| **k** | RF/aCCP |
| **l** | ESR/CRP |
| **Study design** | |
|  | *Item* |
| **m** | Longitudinal(n?) |
| **n** | Blinded |
| **o** | US/CR/CE in short time period at each timepoint |
| **p** | definition diagnosis |
| **q** | definition remission |
| **r** | definition of erosive disease |
| **s** | Definition flare (if applicable) |
| **t** | currently known covariates recorded |
| **Analysis** | |
|  | *Item* |
| **u** | Multivariate logistic regression |
| **v** | Number of cases >=10 per covariate |
| **w** | RR/OR +- 95%CI |
| **x** | Definitions US inflammation clear |
| * The quality of each parameter is described as “Good” when present, “Bad” when absent or “Unclear” when it is not clear if the parameter is present. | |
